# Cognitive aids for people with early stage dementia versus treatment as usual (Dementia Early Stage Cognitive Aids New Trial (DESCANT)): study protocol for a randomised controlled trial

**DOI:** 10.1186/s13063-018-2933-8

**Published:** 2018-10-10

**Authors:** Helen Chester, Paul Clarkson, Linda Davies, Jane Hughes, Muhammad Saiful Islam, Narinder Kapur, Martin Orrell, Julie Peconi, Rosa Pitts, Fiona Poland, Ian Russell, David Challis, Members of the HoSt-D (Home Support in Dementia) Programme Management Group

**Affiliations:** 1 Personal Social Services Research Unit, Division of Population Health, Health Services Research and Primary Care, School of Health Sciences, Faculty of Biology, Medicine and Health, University of Manchester, Manchester Academic Health Science Centre, Manchester, UK; 2 Manchester Centre for Health Economics, Division of Population Health, Health Services Research and Primary Care, School of Health Sciences, Faculty of Biology, Medicine and Health, University of Manchester, Manchester Academic Health Science Centre, Manchester, UK; 3 Swansea Trials Unit, Institute of Life Science 2, Medical School, Swansea University, Swansea, UK; 4 Research Department of Clinical, Educational and Health Psychology, University College London, London, UK; 5 Institute of Mental Health, School of Medicine, University of Nottingham, Nottingham, UK; 6 School of Health Sciences, Faculty of Medicine and Health Sciences, University of East Anglia, Norwich, UK

**Keywords:** Early-stage dementia, Memory aids, Community, Psychosocial outcomes, Quality of life, Effectiveness, Cost-effectiveness, Pragmatic randomised trial, Process evaluation

## Abstract

**Background:**

There is a growing need for an evidence-based approach to home support for people with dementia and their carers following diagnosis but research on the effectiveness and cost-effectiveness of different approaches is sparse. The Dementia Early Stage Cognitive Aids New Trial (DESCANT) will evaluate the clinical and cost-effectiveness of a range of memory aids, training and support to people with mild to moderate dementia and their carers at home and compares that intervention with treatment as usual.

**Methods/design:**

This is a multi-site, pragmatic randomised trial preceded by a feasibility study and internal pilot. We aim to allocate at random 360 pairs comprising a person with mild to moderate dementia and an identified carer between the DESCANT intervention and treatment as usual. We assess participants at baseline, 13 and 26 weeks. The primary outcome measure is the Bristol Activities of Daily Living Scale; other participant outcomes include cognition, quality of life, activities of daily living and social networking; carer outcomes include quality of life, sense of competence and mental health. To enhance this quantitative evaluation we are conducting a qualitative component and a process evaluation to assess the implementation process and identify contextual factors associated with variation.

**Discussion:**

The DESCANT intervention reflects current policy to enhance the capabilities of people with dementia after diagnosis and their carers. If it is clinically and cost-effective, its modest nature and cost will enhance the likelihood of it being incorporated into mainstream practice.

**Trial registration:**

Current Controlled Trials, ISRCTN12591717. Registered on 29 July 2016.

Protocol number**:** 31288: North West - Haydock Research Ethics Committee, 20/06/2016, ref.: 16/NW/0389.

## Background

Worldwide there were about 47 million people living with dementia in 2015; since there is no cure the costs associated with the condition will increase [1]. In England there is considerable interest in achieving early diagnosis and treatment of dementia through memory services, and providing a spectrum of care including interventions and support after diagnosis [2–4]. Diagnosis can help people with dementia and their carers to receive the treatment, care and support (pharmacological, psychosocial, social and emotional) to enable them to manage this condition.

Rigorous research is required to inform the choice of treatment options [5]. However robust evaluation of the use of common memory aids, like calendars, clocks, whiteboards with electric timers and “post-it” note dispensers, by those diagnosed with early dementia is lacking despite being widely recommended [6, 7] . A Cochrane review [8] identified several studies that reported the utility of memory aids or associated memory training, but they were small scale, highlighting the need for a larger study [9–11].

This trial aims to design, implement and evaluate an intervention to support people with early-stage dementia and their carers in the use of memory aids at home. It is one of nine distinct, but linked, projects within a research programme designed to discern different models of support in England and evaluate cost-effectiveness in providing care for people with dementia and their carers at home; the Personal Social Services Research Unit (PSSRU) at the University of Manchester is leading this trial.

**Fig. 1:**
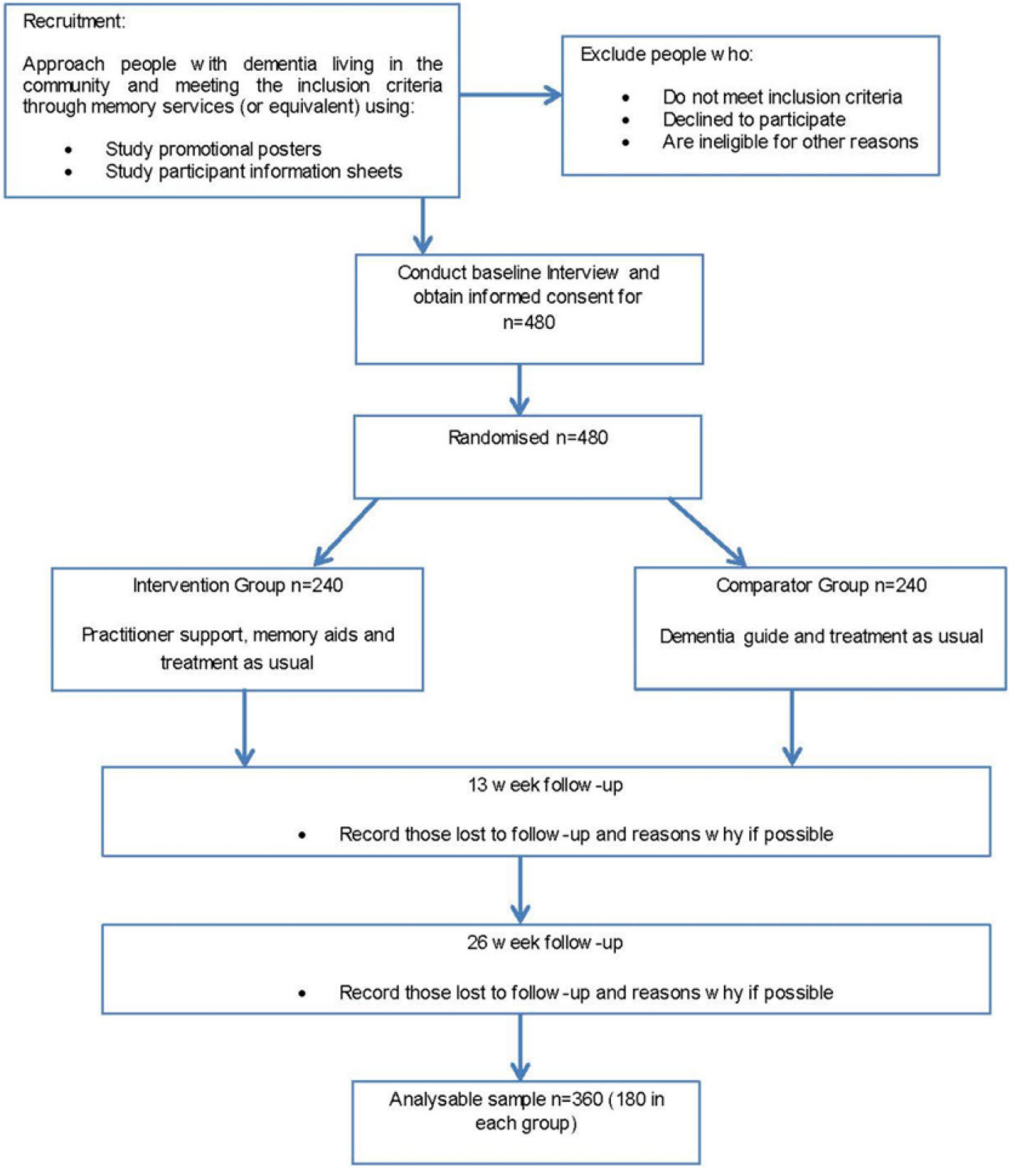
DESCANT trial flowchart

Figure 1 shows a Consolidated Standards of Reporting Trials (CONSORT) flowchart of the trial.

## Methods/design

### Governance arrangements

The Programme Steering Committee (PSC) oversees the programme of which this trial is part. It comprises representatives of patients and public, old age psychiatry, psychology, health services research and biostatistics, and works to an agreed Charter. It also acts as the independent Trial Steering Group.

This trial has been adopted by the Swansea Trials Unit (STU) and will be conducted according to its standard operating procedures. The Trial Management Group comprises PSSRU staff at the University of Manchester, and it monitors compliance and reports to NHS Trusts, oversees and resolves operational issues in partnership with colleagues at STU, and reports to the Data Monitoring and Ethics Committee (DMEC) and the funder, the National Institute of Health Research (NIHR). The DMEC meets at least annually and reports to the PSC; the chair is a geriatrician with a particular interest in dementia, and their charter reflects established practice [12]. Only the DMEC have access through the Trial Data Manager to unblinded data before the trial ends; it will unblind the DESCANT analysis team only after approving the blinded primary analysis.

The Public, Patient and Carer Reference Group (PPCRG) comprises a regular meeting of 6 members resident in North West England and a Lay Advisory Panel of 20 members across the country, established through Together in Dementia Everyday (TIDE).

### Design

This is a multi-site, pragmatic randomised trial to evaluate the effectiveness and cost-effectiveness of memory aids delivered by Dementia Support Practitioners (DSPs) to people with early-stage dementia relative to treatment as usual (TAU). Pairs of people with mild to moderate dementia and an identified carer will be recruited and allocated at random between the comparator group receiving TAU plus dementia guide [13] and the intervention group receiving TAU plus the DESCANT intervention. The primary outcome measure is the Bristol Activities of Daily Living Scale (BADLS) [14], measured at 13 and 26 weeks (primary end point) after baseline.

To ensure intervention and methods work in practice, a feasibility study and internal pilot was conducted with 40 participants, recruited from two settings in equal numbers. The Acceptance Checklist for Clinical Effectiveness Pilot Trials (ACCEPT) criteria [15] was adapted to assess whether the intervention and the trial protocol work in practice, and the DMEC had accepted these criteria. The only adjustments suggested by the pilot was to reduce the length of follow up from 12 to 6 months in the main study to enable the recruitment targets to be met and reflect the realistic goal of improving BADLS scores over 6 months. Therefore, the pilot data will be included, adjusted to reflect this change in the main analysis. This modification, approved by the University of Manchester as Sponsor, was communicated to the National Research Ethics Service who approved the original protocol (reference 15/NW/0822), sites and participants.

### Intervention

**Fig. 2:**
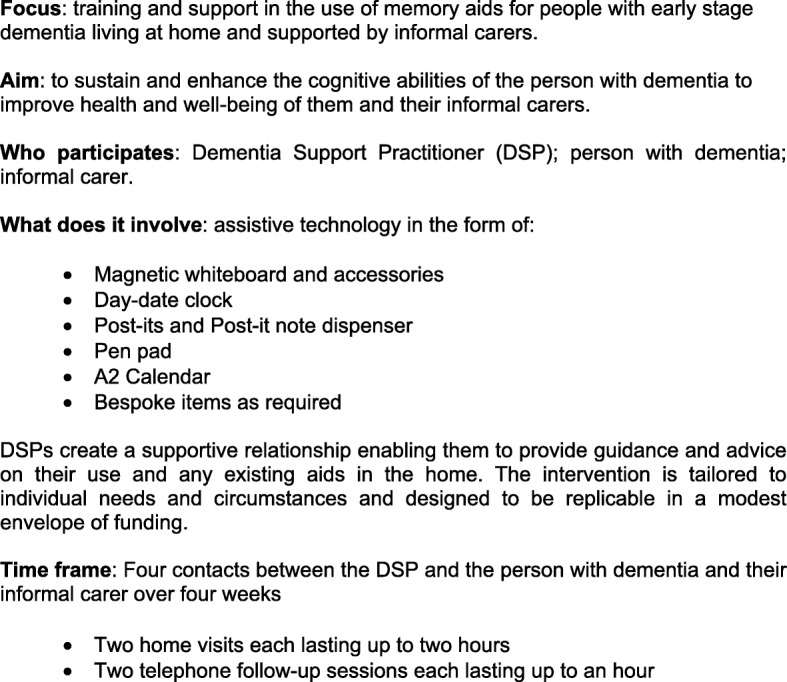
DESCANT intervention. Source: [62]

The manualised intervention adds specialist equipment and advice by trained DSPs to TAU. It provides up to 6 hours contact with a DSP for the person with dementia and the identified carer (jointly referred to as participants). DSPs design and deliver a package of memory aids up to a maximum of £150 for the person with dementia to use at home. The package for each depends on their needs, preferences and existing use of memory aids. DSPs also advise on improving everyday memory skills and on using these aids to reduce memory lapses. The follow-up sessions address queries from participants and record whether aids are appropriate to the identified goals and needs and used accordingly. Further details about the implementation of the intervention are provided in Fig. 2.

#### Treatment as usual

TAU comprises help from memory clinic staff, post diagnostic counselling and advice, and specialist follow up as appropriate. Participants in the comparator group also receive a general guide to dementia for patients and carers [13].

### Setting

The study runs in three National Health Service (NHS) Trusts in England, and more may follow. To be eligible participating Trusts must provide a memory service, a health-based resource providing early diagnosis and support for people with dementia and their carers. They must also identify a local Principal Investigator (PI), obtain support from their local Research and Development department and Clinical Research Network (CRN) to complete the research activities described in this protocol, and employ appropriately experienced persons as DSPs.

Participants will be recruited primarily from memory services. “Join Dementia Research,” an on-line self-registration service enabling volunteers with memory problems or dementia and their carers to register their interest in taking part in research, will also be used as a recruitment tool.

### Participants

#### Inclusion criteria

Index people with dementia must be aged 50 years or more; cared for by a trial memory clinic or equivalent; within one year of their first attendance at that clinic for dementia; physically able to engage with the intervention (as judged from clinical records); clinically able to do so, as judged by a responsible clinician and have dementia of mild to moderate severity. At baseline, they must live in their own home or share a home with a relative. They must have an identified carer, defined as the primary person who feels responsible for and supports them. This could be a family member, a close friend or a neighbour.

### Recruitment

#### Sites

Sites will be recruited via professional and research networks, including the UK NIHR Clinical Research Network (CRN). Participating Trusts will identify memory clinics for inclusion in the study. Each site will confirm their capacity to undertake the trial by completing the Health Research Authority Statement of Activities as a formal agreement with the Sponsor.

#### People with dementia and their carers

Clinical staff in participating Trusts are briefed about the trial and provided with participant information sheets (PISs) for potential participants. They will complete a screening schedule to check eligibility, give the PIS to participants, and seek oral consent to refer them to the DESCANT team. Recruitment, documents and processes were designed following guidance from the PPCRG on language and format, to ensure that those with cognitive impairment are fully informed and engaged in the decision to take part.

#### Consent process

Clinical staff within agencies will introduce the study to participants, provide them with PISs and obtain their consent to contact by a member of the research team. Before conducting the interview, researchers explain the study to participants and obtain their formal consent. Guided by the Mental Capacity Act 2005 [16], it is judged that people with mild to moderate dementia approached in this way have capacity to provide informed consent, given sufficient time to decide.

Participants may withdraw from the trial at any time for any reason. If they withdraw, a reason will be recorded unless they choose not to provide one. PIs may also withdraw people with dementia from the trial if they feel it is no longer in their best interest to continue. Unless participants request otherwise, data collected before withdrawal will be retained for analysis. Each site maintains a log of people who satisfy the inclusion criteria but are not recruited, including demographic details and reasons for not consenting to participate if given.

### Outcome measures

**Fig. 3:**
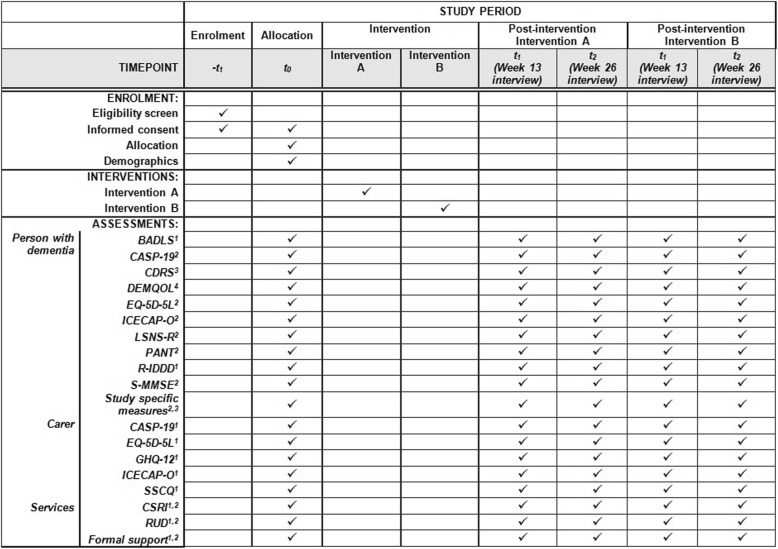
Schedule of enrolment, interventions and assessment points. BADLS: Bristol Activities of Daily Living Scale; CASP-19: Control, Autonomy, Self-realisation and Pleasure 19-item measure of quality of life; CDRS: Clinical Dementia Rating Scale; DEMQOL: Dementia Quality of Life scale; ICECAP-O: ICEpop (Investigating Choice Experiments for the Preferences of Older People) CAPability measure for Older people; LSNS-R: Lubben Social Network Scale - Revised; PANT: Practitioner Assessment of Network Type; R-IDDD: Revised Interview for Deterioration in Daily living activites in Dementia; S-MMSE: Standardised Mini-Mental State Examination; GHQ-12: General Health Questionnaire; SSCQ: Short Sense of Competence Questionnaire; CSRI: Client Service Receipt Inventory; RUD: Resource Utilisation in Dementia. Completion by 1 = carer; 2 = person with dementia; 3 = interviewer; 4 = either person with dementia or carer. Intervention A = Comparator group; Intervention B = Intervention Group

The schedule of outcome measures and assessment points are provided in Fig. 3.

#### Primary outcome measure

##### Bristol Activities of Daily Living Scale

This assesses the independence of the person with dementia in activities of daily living [14]. As they may not always be aware of their deficits, carers assess these. The measure captures basic and instrumental abilities including dressing, bathing, food preparation and use of the telephone. It is valid and reliable and responsive to change over time [17]. Therefore, it is appropriate to evaluate a practical intervention designed to extend a person’s competence in daily living.

#### Secondary outcome measures for persons with dementia

##### Cognition (two measures)

The Standardised Mini-Mental State Examination (S-MMSE) is a brief widely used self-completed test of cognitive function with good validity and reliability, which measures the severity of cognitive symptoms of dementia [18]. The Clinical Dementia Rating (CDR) is an interviewer-completed scale to indicate level of impairment in six domains [19].

##### Quality of life (three measures)

Investigating Choice Experiments for the Preferences of Older People Capability measure for Older people (ICECAP-O) measures quality of life in older people across five domains [20]. The control, autonomy, self-realisation and pleasure 19-item measure (CASP-19), measures quality of life in older people across another four domains [21, 22]. Both these measures are self-completed by the person with dementia. The Dementia Quality of Life scale (DEMQOL) measures five domains of quality of life in dementia and has good validity and reliability [23]; either the person with dementia completes this or the carer completes a proxy version.

##### Activities of daily living

The Revised Interview for Deterioration in Daily living activities in Dementia (R-IDDD) enables carers to rate patients’ initiative and performance of daily living activities [24, 25].

##### Social network (two measures)

The Lubben Social Network Scale - Revised (LSNS-R) is designed to measure social isolation in older adults through perceived support from family and friends [26]. The Practitioner Assessment of Network Type (PANT) [27, 28] allocates participants to one of five types of network reflecting their contact with family, friends and neighbours. The person with dementia completes both measures.

#### Secondary outcome measures for carers

##### Quality of life (two measures)

Carers complete both the ICECAP-O [20] and CASP-19 [21, 22] as summarised above.

##### Sense of competence

The Short Sense of Competence Questionnaire (SSCQ) enables carers to assess their own competence to cope with the person with dementia [29].

##### Mental health

The self-completed General Health Questionnaire (GHQ-12) will be used to identify minor psychiatric morbidity in carers [30].

#### Economic measures

##### Resource use (two measures)

The Client Service Receipt Inventory (CSRI) is a template used extensively in studies of mental health and dementia to record details of formal services received [31]. This is supplemented by study-specific measures of participants’ use of equipment, adaptations and ambulances (formal support). The Resource Utilisation in Dementia questionnaire (RUD) complements the CSRI by estimating the volume, duration and cost of support from formal and informal carers. People with dementia and carers complete both measures [32].

##### Health-related quality of life

The Euroquol 5-dimension 5-level questionnaire (EQ-5D-5 L) provides a simple descriptive profile that generates a single utility value for health status to assess quality of life [33, 34]. The person with dementia and the carer completes this scale.

#### Other data collected

##### Socio-demographic information

Interviewers collect socio-demographic data from both the person with dementia and the carer.

##### Study-specific measures

Interviewers collect data on comorbidities in the person with dementia, current use of memory aids and current medication. They also assess how confident they are about participants’ allocation to the trial groups.

##### Serious adverse events

In accordance with Good Clinical Practice all data collectors are encouraged to report deaths and adverse events that are life threatening, require or extend hospitalisation, result in disability or incapacity or are otherwise significant.

### Procedure

#### Data collection

Interviewers, who are masked to the allocated group, interview participants face to face at home at baseline and 13 and 26 weeks after first interview. Data quality will be promoted in two ways: interviewers undertake online training (focussing on completing outcome measures) and sites check all documents to minimise missing data.

#### Random allocation

The Trial Manager (TM) coordinates recruitment from all sites and forwards details to STU through their email-based randomisation service. After baseline interviews, the unmasked Trial Data Manager (TDM) at STU oversees stratification of people with dementia by Trust, age, gender, whether living with a carer and time since first attendance at memory clinic or equivalent. Allocation between intervention and comparator groups using dynamic software randomises participants in real time, thus preventing subversion while ensuring (stochastic) balance between the two groups [35].

The person with dementia receives a letter specifying their allocated group and reminding them what this entails. Those in the comparator group receive a general guide to dementia [13]. The intervention group receive confirmation of arrangements for an initial visit by the DSP after about 2 weeks.

### Masking

Interviewers, statisticians, economists, PSC and DMEC will be masked to participants’ allocations between the two groups. However, masking participants and DSPs is not possible. To minimise the risk of bias [36], several precautions are taken:

1. The fieldwork manual for interviewers and support staff at each site explains the case for masking and its importance.
2. Site coordinators are asked to deny interviewers access to allocations, for example in the recruitment log.
3. All staff are asked to minimise participant-specific discussion.
4. Deliberate disclosure of participants’ allocations is limited to serious adverse events or safeguarding issues.
5. All cases of unmasking - by participants, interviewers, clinical or support staff or researchers - are recorded, including the reason for unmasking.
6. Masked interviewers record after each interview to which group they judge the pair belong and with how much confidence.
7. Statistical tests will be undertaken to determine whether, and if so how, unmasking implicitly or explicitly affects estimated parameters.

### Data management

Personal information will be held securely in accordance with data protection legislation. Screening forms completed by clinic staff and questionnaires completed by interviewers will be transferred to the TM by encrypted NHSmail. Unmasked researchers supervised by the TDM based at STU enter data into MACRO4 electronic data collection system provided by Elsevier (2017) [37] in accordance with the study-specific handbook [38]. Participants’ trial numbers will be used to link their data and ensure they remain anonymous. A full audit trial will be maintained by recording all amendments to data with the reason and the time of amendment. Thus, the Sponsor may also inspect or audit the study at any time. The STU will compile the final trial dataset in MACRO4 and transfer it to the PSSRU only when it is complete.

### Sample size

An analysable sample of 360 (180 in each group) across participating Trusts would yield 80% power to detect an effect size (standardised mean difference) of 0.30 on BADLS when using a two-sided significance level of 5%. To allow for 25% attrition (estimated from previous similar studies) between baseline and final interviews the aim is to recruit 480 randomised pairs of people with mild to moderate dementia and an identified carer.

### Process evaluation

**Fig. 4:**
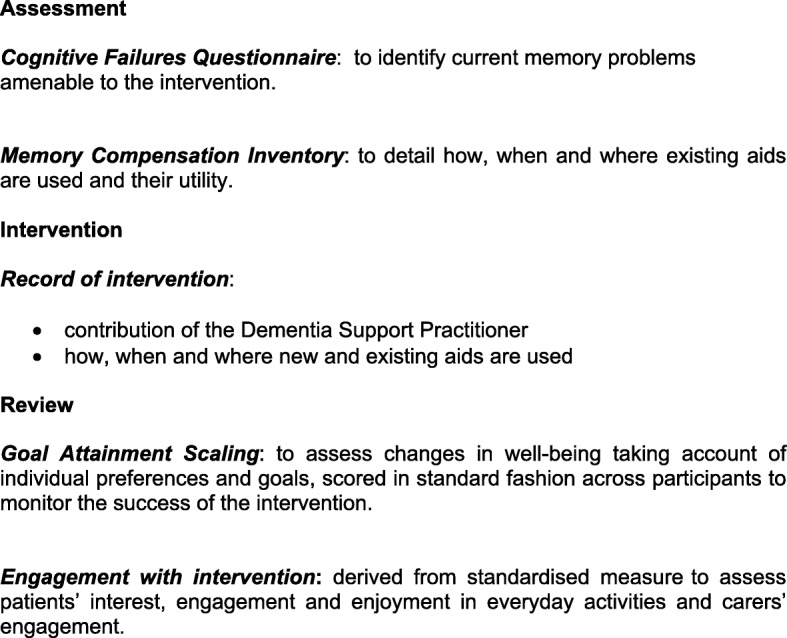
Process evaluation framework for intervention group

The Medical Research Council advocates process evaluations within trials of complex interventions to assess implementation and identify contextual factors associated with variation in outcomes [39]. To replicate the DESCANT intervention will need understanding of how it works in the intervention group. Figure 4 shows the process evaluation framework comprising a mix of standardised [40–42] and study-specific measures. Data are also collected from participants about appropriateness and timeliness and from DSPs about barriers and facilitators to implementation and future roll-out.

### Qualitative data collection

To enhance evaluation of its intervention, DESCANT includes a qualitative component. Audio-recorded structured interviews of a sub-sample of participating pairs will provide richer contextual and conversational data about participants’ experiences and assessments of the role of aids in combating memory loss. These data will be collected unobtrusively at baseline interview [43].

Participants will be included from the intervention and comparator groups to avoid unmasking of interviewers by the research team and ensure potential bias from audio-recording affects each group equally. The sample size will be set according to practical criteria like available resources and time and when data saturation is achieved [44]. It is predicted that this will need a judgement sample of at least 15 participants identified sequentially from the baseline sample.

### Statistical analysis

Analysis will follow a defined statistical analysis plan approved by the DMEC before data are accessed. Baseline characteristics of participants will be presented. Continuous variables will be reported by means and standard deviations and categorical variables by counts and percentages. Analyses by treatment allocated will estimate the effect of the intervention on participants, using analysis of covariance to adjust for baseline differences in demographic characteristics, comorbidities, time since first attendance at memory clinic or equivalent and for the interval until follow up. Primary analysis will focus on BADLS scores at 26 weeks. Secondary analysis will take account of the content of, and adherence to, the intervention to explore the effect of compliance on BADLS scores.

To explore whether people with dementia who have particular characteristics are more likely to use specific aids, logistic regression will be used. To identify which participants are more likely to benefit from this intervention, linear regression will be used to relate change in BADLS scores at 26 weeks to similar covariates.

Whether the inevitable missing data cause bias will be investigated. If predictors of missing data are identified that are related to outcomes, multiple imputation to adjust for these biases will be used.

#### Economic evaluation

Cost-effectiveness analysis will be undertaken to evaluate the full cost of this intervention and whether it yields value for money. National unit costs for specific items of service will be used to estimate the costs of services used. Primary analysis will estimate quality-adjusted life years (QALYs) from the EQ-5D-5 L using the value set recommended by the National Institute for Health and Care Excellence (NICE) at the time of the analysis [45]. Current value sets include the EQ-5D-5 L specific utility weights [46] and the crosswalk algorithm to map the EQ-5D-5 L survey to the EQ-5D-3 L value set [47]. QALYs gained between baseline and follow-up interviews (13 and 26 weeks) will be estimated as the number of weeks multiplied by the utility of observed survival. Covariates that affect costs or outcomes from relevant published economic evaluations will be identified [48]. Adjusted costs and QALYs will be bootstrapped to estimate pairs of net costs and QALYs. These will then be used to estimate the probability that the intervention may be cost-effective. Net benefit will be estimated by valuing net QALYs to reflect decision makers’ willingness to pay (WTP) for one QALY. Recent decisions by the NICE suggest that WTP is at most £20 k [49, 50]. Cost-effectiveness planes and cost-effectiveness acceptability curves (CEACs) will be plotted to summarize uncertainty associated with incremental cost-effectiveness ratios by the probability that the intervention in question is cost-effective [51].

This economic model will be varied to explore uncertainty arising from study design decisions by using higher and lower sets of unit costs; an earlier estimate of utility from the EQ-5D-5 L [47]; BADLS, the primary outcome measure, or the DEM-QoL to measure health benefit; different approaches to missing data (for example by analysing only complete cases) and predicting costs and QALYs over longer time horizons (for example 5 and 10 years).

#### Process data analysis

This analysis will use the framework in Fig. 2. Again, continuous variables will be reported by means and standard deviations and categorical variables by counts. Both standardised and study-specific measures will need analysis of covariance to identify predictive factors.

#### Qualitative data analysis

Thematic and narrative analysis of interview transcripts will be undertaken to elicit the experiences of people with dementia and their carers. At least two researchers will analyse each transcript. Once all issues have been identified, an iterative process will be used to identify the major themes and processes through conceptual abstraction. Achievement of data saturation will be informed by a focus group of interviewers [52].

## Discussion

This programme of work focusses on formal support at home from health and social services to complement informal support provided by family and friends. It includes: supportive practical and emotional help; structured therapeutic interventions, such as counselling; and education, including training in managing behaviour [53]. This trial was designed to fill a knowledge gap identified by preceding systematic review [54]. It also took account of another recent study within the programme, which showed that people with dementia prefer advice on memory aids to be provided at home by a trained worker [55].

There is little evidence on the effectiveness and cost-effectiveness of support for people with dementia and their carers [56–58]. Nevertheless, English health and social care policy continues to emphasise the importance of early diagnosis and support for people with dementia living in their own homes [59, 60]. This assumes that this will improve the quality of life of people with dementia. If so this needs an evidence-based framework for a national pathway with an affordable implementation plan to improve the quality of post-diagnosis dementia care [60].

There are benefits to health and social care from knowing what forms of home support enhance patients’ experience and management of symptoms, over what is currently available. The trial will contribute to this evidence base if the intervention is clinically effective and cost-effective. It is hypothesised that by supporting the cognitive abilities of people with early dementia, memory aids, and training and support in their use, may improve their function, health and well-being and that of their carers. This trial will therefore evaluate the effects on people with dementia and their carers of this modest intervention, designed to be cheap, realistic and scalable [61].

It is planned to present findings to professionals who care for people with dementia; and to submit them for publication in journals reporting research into assistive technologies. These findings will also inform other studies within the programme, notably, economic modelling of the effects of different home support packages and the development of a toolkit for managers and commissioners, likely to be publicly available online with one module dedicated to findings from this trial.

### Trial status

This trial is currently recruiting. Recruitment started in November 2016 (first enrolment 6 December 2016) and is due to continue through 2018.

## Abbreviations

ACCEPT: Acceptance Checklist for Clinical Effectiveness Pilot Trials
BADLS: Bristol Activities of Daily Living Scale
CASP-19: Control, Autonomy, Self-realisation & Pleasure 19-item measure of quality of life
CDR: Clinical Dementia Rating scale
CEAC: Cost-effectiveness acceptability curves
CONSORT: Consolidated Standards of Reporting Trials
CRN: Clinical Research Network
CSRI: Client Service Receipt Inventory
DEMQOL: Dementia Quality of Life measure
DESCANT: Dementia Early Stage Cognitive Aids New Trial
DMEC: (Trial) Data Monitoring and Ethics Committee
DSP: Dementia Support Practitioner
GHQ: General Health Questionnaire
HoSt-D: Home Support in Dementia
ICECAP-O: Investigating Choice Experiments: Capability measure for Older people
LSNS-R: Lubben Social Network Scale – Revised
NHS: National Health Service
NIHR: National Institute for Health Research
PANT: Practitioner Assessment of Network Type
PI: Principal Investigator
PIS: Participant information sheet
PPCRG: Public, Patient and Carer Reference Group
PSC: Programme Steering Committee
PSSRU: Personal Social Services Research Unit
QALYs: Quality-Adjusted Life Years
R-IDDD: Revised Interview for Deterioration in Daily living activities in Dementia
RUD: Resource Utilisation in Dementia
S-MMSE: Standardised Mini-Mental State Examination
SSCQ: Short Sense of Competence Questionnaire
STU: Swansea Trials Unit
TAU: Treatment as usual
TDM: Trial Data Manager
TIDE: Together in Dementia Everyday
TM: Trial Manager
TSC: Trial Steering Committee
UCL: University College London
WTP: Willingness to pay
